# Supplementary material for: Autonomous Purkinje cell activation instructs bidirectional motor learning through evoked dendritic calcium signaling
Source: Nat Commun. 2021 Apr 12;12:2153. doi: 10.1038/s41467-021-22405-8 (PMC8042043; doi:10.1038/s41467-021-22405-8)
Supplement: Supplementary file 1 — Supplementary Information [file 41467_2021_22405_MOESM1_ESM.pdf]

Supplementary Information

Autonomous Purkinje cell activation instructs bidirectional motor learning through evoked dendritic calcium signaling

Audrey Bonnan[1], Matthew M.J. Rowan[1,2], Christopher A. Baker[1],
M. McLean Bolton[1] and Jason M. Christie[1,3*]

[1]Max Planck Florida Institute for Neuroscience
1 Max Planck Way
Jupiter, FL 33458

[2]Current Address: Emory University School of Medicine
615 Michael Hall Street
Atlanta, GA, 30322

[3]Current Address: University of Colorado School of Medicine
12800 East 19th Avenue
Aurora, CO 80045

Includes Supplementary Figures 1-6 and Supplementary Table 1.

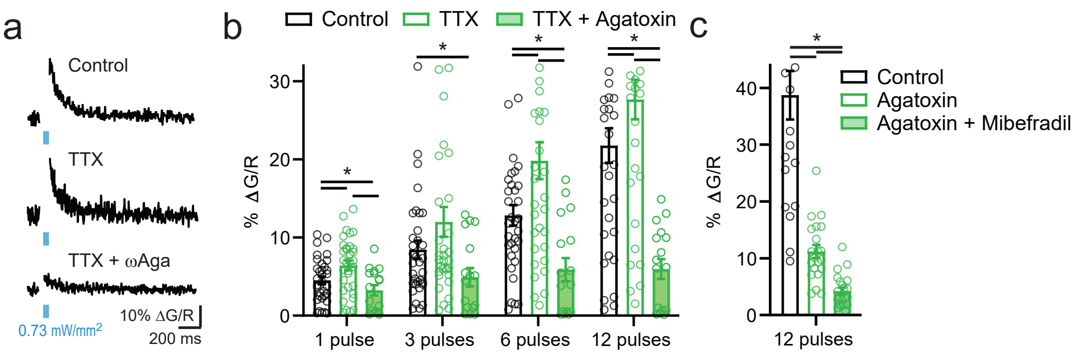

Supplementary Figure 1 | Ca^{2+} channels contribute to optogenetically evoked Ca^{2+} signaling in PC dendrites.

(a) In the same PC, dendritic Ca^{2+} transients evoked by high-power light stimuli (λ 461 nm; 3 pulses, 5 ms, 50 Hz) in control, TTX (1 μM), or TTX and ω-agatoxin (0.5 μM).
(b) Within PC comparison (n = 33 dendrites from 7 PCs for control and TTX, 6 mice; n = 17 dendrites from 4 PCs for TTX + ω-agatoxin, 4mice) shows optogenetically evoked Ca^{2+} signals are reduced by P/Q-type Ca^{2+} channel block ($p < 0.001$; 2-way ANOVA with Tukey's post-test; 1 pulse : control vs. TTX, p = 0.0006, control vs.TTX+ ω-agatoxin, p = 0.0088, TTX vs. TTX+ ω-agatoxin, p < 0.0001; 3 pulses : control vs. TTX, p = 0.069, control vs.TTX+ ω-agatoxin, p = 0.0135, TTX vs. TTX+ ω-agatoxin, p = 0.0564; 6 pulses : control vs. TTX, p = 0.0082, control vs.TTX+ ω-agatoxin, p < 0.0001, TTX vs. TTX+ ω-agatoxin, p = 0.0012; 12 pulses : control vs. TTX, p = 0.0033, control vs.TTX+ ω-agatoxin, p < 0.0001, TTX vs. TTX+ ω-agatoxin, p < 0.0001).
(c) The inhibitory effect of P/Q Ca^{2+} channel block alone on optogenetically evoked Ca^{2+} transients to repeat high-intensity stimulation (n = 21 dendrites from 4 PCs; 3 mice). The addition of T-type Ca^{2+} channel blockade further reduced the response ($p = 0.0003$; 1-way ANOVA with Tukey's post-test).
All data are mean ± SEM; asterisk indicates $p < 0.05$.

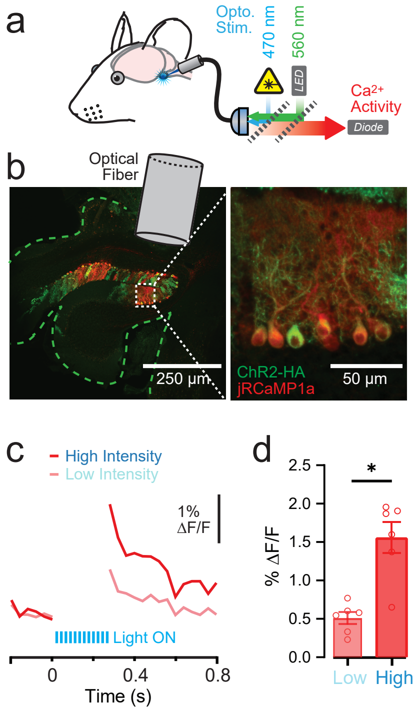

Supplementary Figure 2 | In vivo Ca^{2+} signaling evoked by PC optogenetic activation.

(**a**) An implanted optical fiber targeting the flocculus was used to simultaneously photoactivate and measure Ca^{2+} activity in PCs.

(**b**) Images showing floccular PCs transduced with jRCaMP1a (red) and HA-tagged ChR2, revealed by anti-HA immunostaining (green).

(**c**) Average PC Ca^{2+} responses, measured in an individual mouse, evoked using different powers of light (λ 473 nm ; 12 pulses, 5 ms, 50 Hz; 5 and 30 mW, respectively). Optogenetic stimulus intensity was categorized based on the amplitude of evoked lateral eye movements during quiescence (see Supplementary Figure 3).

(**d**) Summary plot showing difference in the average size of optogenetically evoked PC Ca^{2+} responses (n = 6 mice). Data are mean ± SEM; asterisk indicates $p < 0.05$; two-tailed paired *t*-test ($p = 0.0007$).

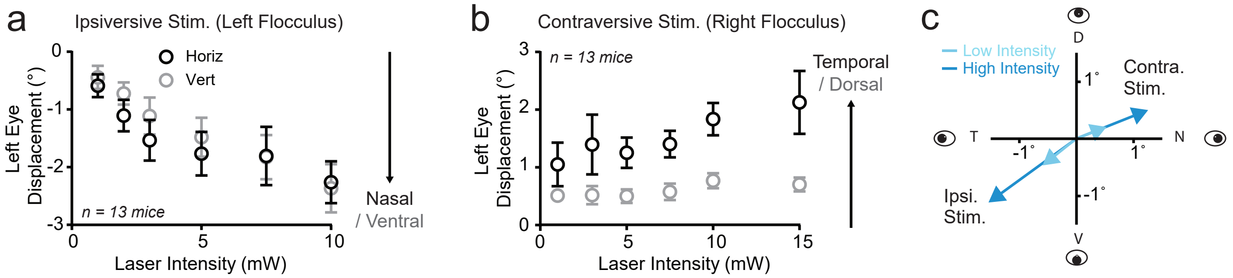

Supplementary Figure 3 | Eye movements evoked by optogenetic PC activation.

(a and b) Decomposed movements of the left eye (lateral displacement in black, vertical displacement in gray) evoked by optogenetically stimulating PCs in the contraversive or ipsiversive flocculus as a function of laser power (λ 473 nm, 12 pulses, 5 ms, 50 Hz) across mice. Data are mean ± SEM.

(c) Eye movement vectors evoked by unilateral contraversive or ipsiversive optogenetic PC activation (T, temporal; N, Nasal; D, dorsal; V, ventral). The amplitude of lateral eye movements was used to categorize the intensity of optogenetic stimuli (< or > 0.75°, low- and high-intensity, respectively).

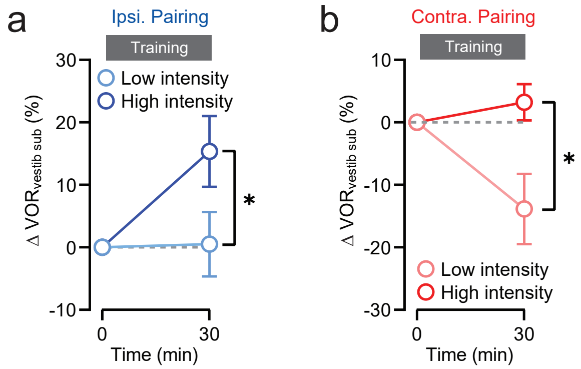

Supplementary Figure 4 | The intensity of PC optogenetic activation determines the VOR learning direction.

(a) Summary plot showing the comparative effect of pairing low- or high-intensity optogenetic PC activation at the end of the ipsiversive phase of vestibular motion on VOR gain. The change attributable to darkness-induced habituation, determined in control training sessions consisting of vestibular motion alone, were subtracted from the test condition to reveal the isolated adaptive effect of the optogenetic stimuli (high intensity, n = 6 mice; low intensity, n = 13 mice; $p = 0.04$; 2-way ANOVA with Sidak's post-test).

(b) For the same mice as above, but comparing the effect of pairing optogenetic PC activation with the end of contraversive vestibular motion (high intensity, n = 6 mice; low intensity, n = 13 mice; $p = 0.02$; 2-way ANOVA with Sidak's post-test).

All data are mean ± SEM.

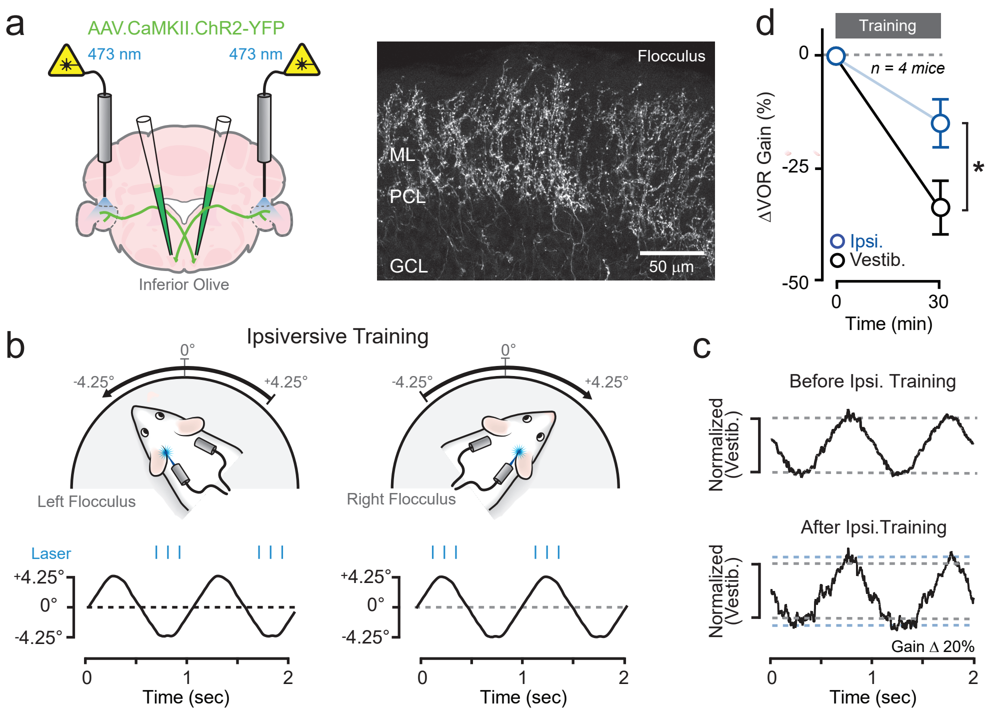

Supplementary Figure 5 | Optogenetic stimulation of climbing fibers induces VOR gain-increase learning.

(a) AAV containing ChR2 under the control of the CaMKII promoter was bilaterally injected into the inferior olive. Optical fiber implants delivered laser light to targeted flocculi. Image shows transduction of YFP-tagged ChR2 in climbing fibers (molecular layer, ML; Purkinje cell layer, PCL; and granule cell layer, GCL).

(b) During training, ChR2-expressing climbing fibers were activated at the end of ipsiversive vestibular motion. In the example traces, the timing of the optogenetic stimulus (3 pulses, 20 ms, 8 Hz) is shown relative to head position.

(c) Average VOR-evoked eye movements before and after repeated pairing of vestibular motion with optogenetic climbing fiber activation. To account for darkness-induced habituation, responses were normalized to a control session consisting of vestibular motion alone.

(d) Effect of optogenetic climbing fiber pairing on VOR gain, shown relative to that of a control training session. Data are plotted as mean ± SEM. Asterisk indicates $p < 0.05$, 2-way repeated measures ANOVA with Sidak's post-test ($p = 0.0162$).

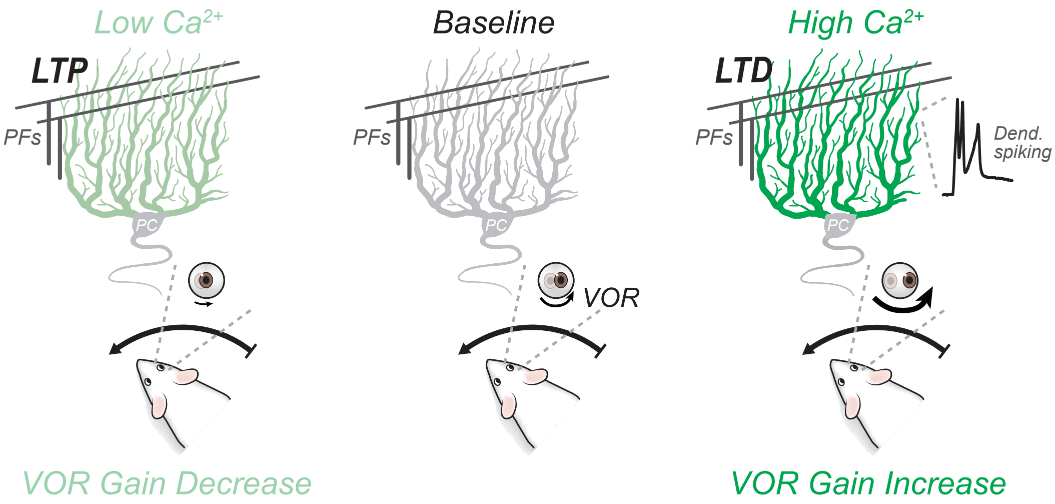

Supplementary Figure 6 | Schematic summary of PC Ca^{2+} signals for instructing bidirectional motor learning.

In the baseline condition, VOR performance engages the activity of parallel fibers (PFs) that impinge onto PCs. When optogenetically evoked Ca^{2+} signals in PC dendrites are repeatedly elicited in conjunction with VOR performance, this drives adaptive changes to the gain of the response. Small-magnitude Ca^{2+} signals, which induce LTP at co-active parallel fiber synapses, results in a decrease in VOR gain. In contrast, large-magnitude Ca^{2+} signals, which are evoked by dendritic spiking and induce LTD at co-active parallel fiber synapses, results in an increase in VOR gain.

Supplementary Table 1

Figure	n (description)	Test Used	*p* value
1e	n = 21 dendrites, from 7 cells, from 6 mice	2way repeated measures ANOVA with Tukey's post-test	1 pulse:<br>- *0.13 vs. 1.68mW p = 0.0345*<br>- *0.13mW vs. CF, p < 0.0001*<br>- *0.73mW vs. CF, p = 0.0404*<br>3 pulses:<br>- *0.13 vs. 0.73mW, p < 0.0001*<br>- *0.13 vs. 1.68mW, p < 0.0001*<br>- *0.13mW vs. CF, p < 0.0001*<br>12 pulses:<br>- *0.13 vs. 0.73mW, p < 0.0001*<br>- *0.13 vs. 1.68mW, p < 0.0001*<br>- *0.13mW vs. CF, p < 0.0001*<br>- *0.73 vs. CF, p = 0.0002*
2b	ChR2, n = 13 cells, from 8 mice<br>Control, n = 8 cells, from 4 mice	Wilcoxon test to compare baseline vs. post conditioning<br><br>Unpaired t-test to compare ChR2 vs. control	ChR2-subthr. : *baseline vs. post cond., p = 0.0005*<br>Control : *baseline vs. post cond., p = 0.9453*<br><br>ChR2 vs. control : *post cond., p = 0.0346*
2d	ChR2, n = 12 cells, from 10 mice<br>Climbing fiber, n = 6 cells, from 4 mice	Wilcoxon test to compare baseline vs. post conditioning<br><br>Unpaired t-test to compare ChR2 vs. control	ChR2-suprathr. : *baseline vs. post cond., p = 0.0049*<br>Climbing fiber : *baseline vs. post cond., p = 0.0313*<br><br>ChR2 vs. control : *post cond., p = 0.0474*
3e	n = 14 mice	2way repeated measures ANOVA with Dunnett's post-test	Vestib. vs. ipsi : *p = 0.6903*<br>Vestib. vs. contra : *p = 0.0388*
3h	n = 7 mice	2way repeated measures ANOVA with Dunnett's post-test	Vestib. vs. ipsi : *p = 0.0068*<br>Vestib. vs. contra : *p = 0.1151*
4b	AM-251, n = 8 cells, from 3 mice<br>Control, n = 7 cells, from 4 mice	Wilcoxon test to compare baseline vs. post conditioning	AM-251 : *baseline vs. post cond., p = 0.4609*<br>Control : *baseline vs. post cond., p = 0.0156*

4c	AM-251, n = 14 cells, from 7 mice<br>Control, n = 12 cells, from 10 mice	Wilcoxon test to compare baseline vs. post conditioning	AM-251 : *baseline vs. post cond., p >0.9999*<br>Control : *baseline vs. post cond., p = 0.0049*
4e	n = 9 mice	2way repeated measures ANOVA with Sidak's post-test	Saline : *contra. vs. vestib., p < 0.05*<br>AM-251: *contra. vs. vestib., p > 0.05*
4f	n = 7 mice	2way repeated measures ANOVA with Sidak's post-test	Saline : *ipsi. vs. vestib., p < 0.05*<br>AM-251: *ipsi. vs. vestib., p > 0.05*
5b	CF, n = 9 cells, from 5 mice<br>ChR2, n = 11 cells, from 5 mice<br>ChR2-Kv2.1, n = 6 cells, from 4 mice	One-way repeated measures ANOVA with Tukey's post-test	CF vs. ChR2: *p > 0.05*<br>CF vs. ChR2-Kv2.1: *p ≤ 0.001*<br>ChR2 vs. ChR2-Kv2.1: *p ≤ 0.001*
5d	n = 25 dendrites, from 4 cells, from 3 mice	2way repeated measures ANOVA with Tukey's post-test	1 pulse : *CF vs. ChR2-Kv2.1, p < 0.0001*<br>3 pulses : *CF vs. ChR2-Kv2.1, p < 0.0001*<br>12 pulses : *CF vs. ChR2-Kv2.1, p < 0.0001*
5f	ChR2-Kv2.1, n = 7 cells from 6 mice<br><br>Control, n = 6 cells from 4 mice	Wilcoxon test to compare baseline vs. post conditioning<br><br>Unpaired t-test to compare ChR2-Kv2.1 vs. control	ChR2-Kv2.1 : *baseline vs. post cond., p = 0.25*<br>Climbing fiber : *baseline vs. post cond., p = 0.0313*<br><br>ChR2 vs. control : *post cond., p = 0.0068*
6c	n = 7 mice	2way repeated measures ANOVA with Dunnett's post-test	Vestib. vs. ipsi : *p = 0.8815*<br>Vestib. vs. contra : *p = 0.3575*
6e	n = 9 mice	2way repeated measures ANOVA with Dunnett's post-test	Vestib. vs. ipsi : *p = 0.4354*<br>Vestib. vs. contra : *p = 0.9509*
6f	n = 6 mice	2way repeated measures ANOVA with Dunnett's post-test	Vestib. vs. ipsi : *p >0.9999*<br>Vestib. vs. contra : *p = 0.9539*
S. 1b	*TTX* : n = 33 dendrites, from 7 cells, from 6 mice<br><br>*TTX+ ω-agatoxin :* n = 17 dendrites, from 4 cells, from 4 mice	2way repeated measures ANOVA with Tukey's post-test	1 pulse :<br>- *control vs. TTX, p = 0.0006*<br>- *control vs.TTX+ ω-agatoxin, p = 0.0088*<br>- *TTX vs. TTX+ ω-agatoxin, p < 0.0001*<br>3 pulses :<br>- *control vs. TTX, p = 0.069*<br>- *control vs.TTX+ ω-agatoxin, p = 0.0135*<br>- *TTX vs. TTX+ ω-agatoxin, p = 0.0564*

			6 pulses :<br>- *control vs. TTX, $p = 0.0082$*<br>- *control vs.TTX+ ω-agatoxin, $p < 0.0001$*<br>- *TTX vs. TTX+ ω-agatoxin, $p = 0.0012$*<br>12 pulses :<br>- *control vs. TTX, $p = 0.0033$*<br>- *control vs.TTX+ ω-agatoxin, $p < 0.0001$*<br>- *TTX vs. TTX+ ω-agatoxin, $p < 0.0001$*
S. 1c	n = 21 dendrites, from 4 cells, from 3 mice	1-way ANOVA with Tukey's post-test	control vs. ω-agatoxin *: $p < 0.0001$*<br>control vs. ω-agatoxin + mibefradil *: $p < 0.0001$*<br>ω-agatoxin vs. ω-agatoxin + mibefradil : *$p = 0.0003$*
S. 2d	n = 6 mice	Paired t-test	High vs. low *: $p = 0.0007$*
S. 4a	High intensity, n = 6<br>Low intensity, n = 13	2way repeated measures ANOVA with Sidak's post-test	High vs. low intensity t_{30} : *$p = 0.0384$*
S. 4b	High intensity, n = 6<br>Low intensity, n = 13	2way repeated measures ANOVA with Sidak's post-test	High vs. low intensity t_{30} : *$p = 0.0164$*
S. 5d	n = 4 mice	2way repeated measures ANOVA with Sidak's post-test	Vestib vs. ipsi stim. : *$p = 0.0162$*
