# Supplementary material for: Autonomous Purkinje cell activation instructs bidirectional motor learning through evoked dendritic calcium signaling
Source: Nat Commun. 2021 Apr 12;12:2153. doi: 10.1038/s41467-021-22405-8 (PMC8042043; doi:10.1038/s41467-021-22405-8)
Supplement: Supplementary file 3 — Reporting Summary [file 41467_2021_22405_MOESM3_ESM.pdf]

nature research

Corresponding author(s): Jason M. Christie

Last updated by author(s): Feb 19, 2021

Reporting Summary

Nature Research wishes to improve the reproducibility of the work that we publish. This form provides structure for consistency and transparency in reporting. For further information on Nature Research policies, see Authors & Referees and the Editorial Policy Checklist .

Statistics

For all statistical analyses, confirm that the following items are present in the figure legend, table legend, main text, or Methods section.

n/a	Confirmed	
☐	☒	The exact sample size (*n*) for each experimental group/condition, given as a discrete number and unit of measurement
☐	☒	A statement on whether measurements were taken from distinct samples or whether the same sample was measured repeatedly
☐	☒	The statistical test(s) used AND whether they are one- or two-sided<br>*Only common tests should be described solely by name; describe more complex techniques in the Methods section.*
☐	☒	A description of all covariates tested
☐	☒	A description of any assumptions or corrections, such as tests of normality and adjustment for multiple comparisons
☐	☒	A full description of the statistical parameters including central tendency (e.g. means) or other basic estimates (e.g. regression coefficient) AND variation (e.g. standard deviation) or associated estimates of uncertainty (e.g. confidence intervals)
☐	☒	For null hypothesis testing, the test statistic (e.g. *F*, *t*, *r*) with confidence intervals, effect sizes, degrees of freedom and *P* value noted<br>*Give P values as exact values whenever suitable.*
☒	☐	For Bayesian analysis, information on the choice of priors and Markov chain Monte Carlo settings
☒	☐	For hierarchical and complex designs, identification of the appropriate level for tests and full reporting of outcomes
☒	☐	Estimates of effect sizes (e.g. Cohen's *d*, Pearson's *r*), indicating how they were calculated

Our web collection on statistics for biologists contains articles on many of the points above.

Software and code

Policy information about availability of computer code

Data collection	Slice physiology data was collected using pClamp (version 2.0.0.10) and PrairieView (version 4.3.2.17) softwares as stated in the Methods section. Data from behavioral experiments was collected using ISCAN eye tracking software (version 1.20Z).
Data analysis	Electrophysiology and calcium imaging data was analyzed using Axograph X (version 1.7.4) and ImageJ (1.52n), respectively. Eye movements and fiberphotometry data was analysed using custom-written Matlab software. Custom Matlab scripts are available upon request.

For manuscripts utilizing custom algorithms or software that are central to the research but not yet described in published literature, software must be made available to editors/reviewers. We strongly encourage code deposition in a community repository (e.g. GitHub). See the Nature Research guidelines for submitting code & software for further information.

Data

Policy information about availability of data

All manuscripts must include a data availability statement. This statement should provide the following information, where applicable:
- Accession codes, unique identifiers, or web links for publicly available datasets
- A list of figures that have associated raw data
- A description of any restrictions on data availability

The data that support this study’s findings are available from the corresponding author upon reasonable request.

Field-specific reporting

Please select the one below that is the best fit for your research. If you are not sure, read the appropriate sections before making your selection.

Life sciences study design

All studies must disclose on these points even when the disclosure is negative.

Sample size	There is no justification of the sample size. Experiments were stopped when the sample size was considered representative according to the literature.
Data exclusions	Cells were excluded from electrophysiology dataset if membrane resistance changed by > 20% during the recording. Animals were excluded from behavioral data ONLY if all conditions for one complete experiment could not be completed (e.g. if one cannula became loose)
Replication	Behavioral training was done in batches of 2 to 4 animals, therefore each figure represents an internally replicated result.
Randomization	For behavioral experiments, the sequence of conditions was randomized for each animal using a random number algorithm. For other experiments, there was no randomization.
Blinding	Data collection and analysis were not blinded. However behavioral data was analyzed using a standardized code to avoid bias.

Reporting for specific materials, systems and methods

We require information from authors about some types of materials, experimental systems and methods used in many studies. Here, indicate whether each material, system or method listed is relevant to your study. If you are not sure if a list item applies to your research, read the appropriate section before selecting a response.

Materials & experimental systems

n/a	Involved in the study	
	x	Antibodies
x		Eukaryotic cell lines
x		Palaeontology
	x	Animals and other organisms
x		Human research participants
x		Clinical data

Methods

n/a	Involved in the study	
x		ChIP-seq
x		Flow cytometry
x		MRI-based neuroimaging

Antibodies

Antibodies used	The following primary antibodies were used : rabbit anti-calbindin (#CB38a, Swant) and rabbit anti-HA (ab9110, abcam)<br>The following secondary antibodies were used : Alexa Fluor-633 goat anti-rabbit (1:1,000 #A-21070, Thermofisher) or Alexa Fluor-488 goat anti-rabbit (1:1000, #A-27034, Thermofisher)
Validation	Rabbit anti-calbindin (#CB38a, Swant) : "This antiserum was produced against recombinant rat calbindin D-28k (CB). It crossreacts<br>with calbindin D-28k from many other species, including human, monkey, rat, mouse chicken<br>and fish. In immunoblots it recognizes a single band of approximately 27 -28 kDa." "Absence of specific immunohistochemical staining with CB 38 in the cerebellum of a Calbindin D28k knock-out mouse." (https://www.swant.com/pdfs/Rabbit_anti_calbindin_D-28k_CB38.pdf)<br>Rabbit anti-HA (ab9110, abcam) : "Immunogen affinity purified." "Positive control : WB: 293FT cells transfected with 15kDa HA tagged Vpr (an HIV1 accessory protein). IP: Nuclear lysate of HEK-293T cells transiently expressing HA-tagged protein. ICC/IF: U-2 cells. Mouse olineu cells. ChIP: Xenopus laevis oocytes were injected with mRNA for HA-tagged human BORIS." (https://www.abcam.com/ha-tag-antibody-chip-grade-ab9110.html)<br><br>Secondary antibodies : :"To minimize cross-reactivity, these goat anti-rabbit IgG (H+L) whole secondary antibodies have been affinity purified and cross-adsorbed against human IgG, human serum, mouse IgG, mouse serum, and bovine serum. Cross-adsorption or pre-adsorption is a purification step to increase specificity of the antibody resulting in higher sensitivity and less background staining. " (thermofischer.com)

Animals and other organisms

Policy information about studies involving animals; ARRIVE guidelines recommended for reporting animal research

Laboratory animals	We used Pcp2::Cre mice (B6.Cg-Tg[Pcp2-Cre]3555jdhu/J; Jax stock #010536). For some experiments, these mice were crossed with the Ai27 reporter line (B6.Cg-Gt[ROSA26]tm27.1-CAG.lsl.ChR2(H134R)-tdTomato;/J; Jax stock #012567). All mice were older than 8 weeks old.
Wild animals	No wild animals were used.

Field-collected samples	No field collected samples were used.
Ethics oversight	All procedure were approved by the Max Planck Florida Institute for Neuroscience IACUC Committee (Institutional Animal Care and Use Committee)

Note that full information on the approval of the study protocol must also be provided in the manuscript.
